# Long-term result of a second or third two-stage revision total knee arthroplasty for infected total knee arthroplasty

**DOI:** 10.1186/s42836-020-00062-4

**Published:** 2021-01-12

**Authors:** Young-Hoo Kim, Jang-Won Park, Young-Soo Jang

**Affiliations:** 1 The Joint Replacement Center of Seoul Metropolitan, SeoNam Hospital, 20, Shinjoung ipen1ro, Yangchun-Gu, Seoul, Republic of Korea; 2 The Joint Replacement Center of Ewha, Womans University Seoul Hospital, Seoul, Republic of Korea

**Keywords:** Long-term result, Second two-stage revision, Third two-stage revision, Infected total knee arthroplasty, Survivorship of TKA

## Abstract

**Background:**

Persistent or recurrent infection after two-stage revision total knee arthroplasty (TKA) for the treatment of an infected TKA is a dreaded complication. The purpose of the current study was to determine the ability of a second or third two-stage revision TKA to control infection, evaluate the long-term survivorship of the TKA prosthesis, and measure the functional outcome after a second or third two-stage revision TKA for reinfection.

**Methods:**

We evaluated 63 patients (65 knees) with failed two-stage TKA treated with a second or a third two-stage revision TKA. There were 25 men and 38 women (mean age, 67 ± 10.2 years). The mean follow-up from the time of a second two-stage TKA revision was 15.1 years (range, 10 to 19 years) and the mean follow-up from the time of a third two-stage TKA revision was 7 years (range, 5 to 10 years).

**Results:**

Overall, infection was successfully controlled in 49 (78%) of 65 knees after a second two-stage revision TKA was performed. In the remaining 16 knees, recurrent infection was successfully controlled in 12 knees (75%) after a third two-stage revision TKA. Survivorship, free of implant removal for recurrent infection, was 94% at 15.1 years (95% CI, 91 to 100%). Survival free of revision TKA for mechanical failure was 95% (95% CI, 92 to 100%).

**Conclusions:**

The results of the current study suggest that a second or a third two-stage revision TKA is a reasonable option for controlling infection, relieving pain, and achieving a satisfactory level of function for patients with infected TKAs.

## Introduction

The reported control rates of infection with two-stage revision total knee arthroplasty (TKA) have ranged from 72–91% [1–9]. Recurrent or persistent infection after two-stage revision TKA for the treatment of an infected TKA is a dreaded complication. Ford *et al*. [1] reported that 30% of patients undergoing two-stage revision TKA had serious complications. There is relatively little literature on the treatment of reinfection following two-stage revision TKA [6, 7, 9, 10]. The optimum treatment of a recurrent infection after two-stage revision TKA remains controversial and varies between patients. Treatment options include antibiotic suppression [11], open debridement [12], resection arthroplasty [13], arthrodesis, staged reimplantation of another prosthesis [14] and amputation [15]. In some patients, one may be inclined to attempt a second or third two-stage revision TKA in an effort to offer more optimal knee function to the patient. Several reports on the outcome of second or third two-stage revision TKA in a small number of patients have shown that it can eradicate the infection and lead to optimal knee function [6, 7, 9, 10, 16].

The purpose of the current study was to: (1) determine the ability of a second or third two-stage revision TKA to control infection; and (2) evaluate the long-term survivorship of a TKA prosthesis and (3) measure the functional outcome after a second or third two-stage TKA for reinfection.

## Patients and methods

**Fig. 1:**
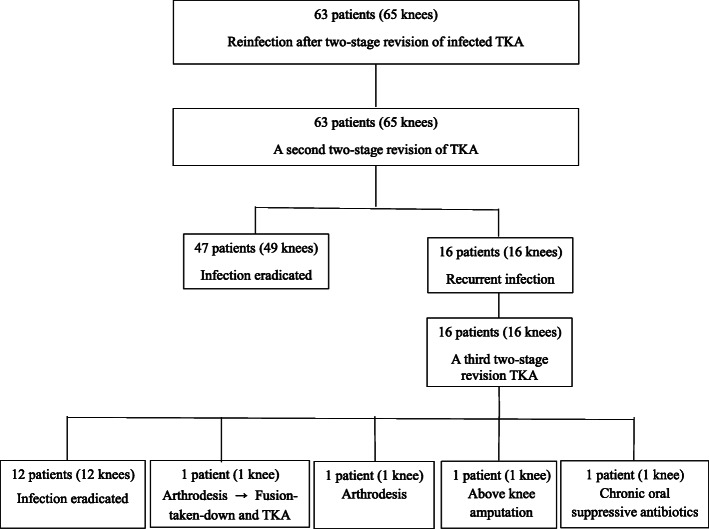
Flow diagram showing the numbers of patients and knees included over the course of the follow-up period

We retrospectively reviewed the database of 66 patients (68 knees). These 66 patients underwent a second two-stage revision TKA between January 2001 and January 2010. Of the 66 patients, 3 (4.5%) were lost to follow-up before 1 year, leaving 63 patients (65 knees) for review. Two patients had bilateral periprosthetic joint infection of the knees and they underwent simultaneous two-stage revision TKAs. The records of 63 patients had been entered into an ongoing computerized database that was updated continuously (Fig. 1). We performed irrigation and debridement after removal of the polyethylene spacer and replaced new polyethylene spacer, with retention of prosthesis as the initial treatment in all patients. Irrigation and debridement failed in all patients undergoing irrigation and debridement, resulting in two-stage revision TKA. There were 25 men and 38 women, with a mean age of 67 ± 10.2 years (range, 40 to 78 years) at the time of a second revision TKA. The mean body mass index was 28.9 ± 2.9 kg/m^2^ (range, 22 to 38.5 kg/m^2^). The study was approved by the institutional review board, and all patients provided written informed consent. The American Society of Anesthesiology (ASA) Score was 2 in 55 patients and 3 in the other 8 patients. Patients were followed at 3 months, 1 year after a second revision TKA and then 2 or 3 years or until a recurrence of infection. The mean follow-up period was 15.1 years (range, 10 to 19 years) after a second two-stage revision TKA and the mean follow-up from time of a third two-stage revision for the 12 TKAs was 7 years (range, 5 to 10 years).

Periprosthetic joint reinfection was diagnosed against the criteria of Musculoskeletal Infection Society (MSIS) [17]. Reinfection was confirmed with positive cultures through aspiration and intraoperative cultures in 59 of the 65 knees (91%) while the other 6 knees met at least one of the 3 criteria: ESR > 20 mm/hr; CRP > 0.5 mg/dL; joint aspiration leukocyte count over 1,100 cells/μL and neutrophil percentage greater than 64%; evidence of purulence during the subsequent surgical intervention [16, 17].

Causative infective organisms included *sStaphylococcus aureus* in 21 knees (32%), methicillin-resistant S*staphylococcus aureus* in 10 (15%), *Staphylococcus epidermis* in 9 (14%), *Streptococcus anginosus* in 7 (11%),*E* *nterococcus cloacae* in 6 (9%), *Candida albicans* in 3 (5%), and *Candida lusitaniae* in 3 (5%). In 6 knees (9%), no organisms were cultured (Table 1). Sixty-two of 65 knees (95%) had the same bacteria and 3 knees (5%) had a different bacteria from the first two-stage revision of TKA; 60 knees (92%) had the same bacteria and 5 knees (8%) had a different bacteria from the second two-stage revision; 13 of 16 knees (82%) had the same bacteria and the remaining 3 knees (18%) had multi-organisms from the third two-stage revision.

All patients underwent removal of all the well-fixed LCCK implants and, mobile bone cement spacer and debridement and placement of a tobramycin-impregnated (1.2 g per 40 g batch of bone cement) mobile cement spacer. Antibiotics were administered intravenously for 6 weeks. After completion of antibiotic therapy, ESR, CRP levels, total WBC count and differential in the joint aspirates and culture from the joint fluid were obtained and the patient was observed for 2 more weeks. If these tests yielded negative results and there was no clinical evidence of recurrent infection (ESR < 20 mm/hr; CRP < 0.5 mg/dl; and joint WBC < 1100 with (64%), we performed a second or third two-stage revision TKA. Multiple cultures of specimens (more than 5 cultures) obtained during a second or third revision operation were performed to confirm negative culture results. The antibiotic-impregnated spacer was removed and Legacy Constrained Condylar Knee prosthesis (LCCK; Zimmer, Warsaw, Indiana) was inserted and fixed with antibiotic-impregnated bone cement (1.2 g tobramycin mixed with 40 g of cement). For fungus infection, amphotericin-impregnated bone cement was used. After reimplantation, antibiotics were stopped at about 2 weeks by recommendation of infectious disease consultant, when the intraoperative cultures were negative, except in one patient in whom chronic oral suppressive antibiotic therapy was used.

At each follow-up, we evaluated the patients clinically and obtained radiographs of knees. Pre-revision and post-revision review data were recorded according to the systems of the Knee Society [18]. All of the knees were evaluated by one orthopedic surgeon who was not connected with the surgery, and the data were entered into a computerized record.

One of the team members evaluated the final radiographs. We defined radiographic loosening as a complete radiolucent line of ≥ 2 mm in width at the bone-cement or prosthesis-cement interface or a shift in position of a component on serial radiographic examination [18].

Descriptive statistics were described as the number (percentage) or mean (range). The chi-square test and Fisher exact test were used to compare binary variables. All calculations assumed 2-tailed test. The level of significance was set at *P* < 0.05. All analyses were performed with SPSS, version 14.0 (SPSS Inc, Chicago, IL).

## Results

Overall, 49 (75%) of 65 knees were survived free of implant removal after a second two-stage revision TKA was performed. The remaining 16 of 65 (25%) knees had a third two-stage revision TKA. At the time of a third two-stage revision TKA, femoral and tibial augmented metallic blocks were used in all of these 16 knees. None of 16 knees required a rotating hinge knee prosthesis. Twelve of 16 knees (75%) undergoing a third two-stage revision TKA had negative culture (Table 1). In four of the 16 knees where infection was not eradicated after a third two-stage revision TKA, one knee had an above-knee amputation, one knee had arthrodesis followed by fusion-taken-down and TKA using an LCCK prosthesis due to intact soft tissue sleeves one year after arthrodesis, one knee had arthrodesis, and one knee received chronic oral suppressive antibiotics because of a poor medical condition.

**Table 1:** Microorganism isolates and control rates of infection after a second two-stage revision TKA

1. *Staphylococcus aureus*	18 of 21 knees (86%)
2. Methicillin resistant *Staphylococcus aureus*	6 of 10 knees (60%)
3. *Staphylococcus epidermidis*	8 of 9 knees (89%)
4. *Streptococcus anginosus*	6 of 7 knees (86%)
5. *Enterococcus cloacae*	4 of 6 knees (67%)
6. *Candida albicans*	1 of 3 knees (33%)
7.* Candida lusitaniae*	1 of 3 knees (33%)
8. No organism	5 of 6 knees (83%)
	49 of 65 knees (75%)

The knees with methicillin resistant *Staphylococcus aureus* or candida organisms tended to have a higher recurrence of infection compared with other organisms. The success rate for combined candida infections was 2 of 6. This is significant when compared all other culture results (2 of 6 versus 47 of 59, *p* < 0.01). Three knees required reoperation for aseptic loosening at a median time of 11.9 years (range, 8.5 to 15.8 years). These knees had negative cultures and negative pathology at the time of a second two-stage TKA. ESR and CRP were within normal the range. At the latest follow-up, all but four components were fixed satisfactorily.

**Fig. 2:**
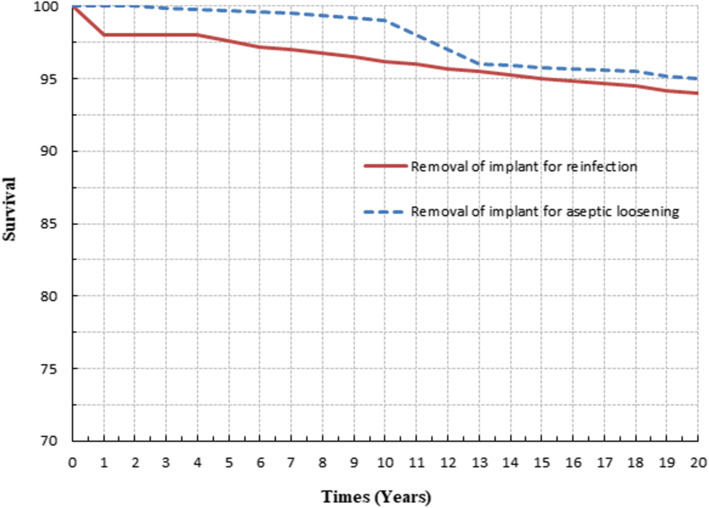
Kaplan-Meier curves show survival rate of the TKA components at 15.1 years with revision TKA due to reinfection or aseptic loosening of the TKA components

The survivorship rate for those knees free of implant removal for reinfection was 94% at 15.1 years (confidence intervals, 91 to 100%). The survival free-of-revision rate for mechanical failure was 95% (confidence intervals, 92 to 100%) at 15.1 years (Fig. 2).

The preoperative Knee Society knee and function scores improved significantly at final follow-up. The preoperative Knee Society knee scores improved (*P* ≤ 0.001) from a median of 50 points (range, 9 to 68 points) to a median of 88 points (range, 61 to 98 points) at the final follow-up. Preoperative functional scores improved (*p* ≤ 0.001) from a median of 7 points (range, 0 to 80 points) to a median of 55 points (range, 15 to 100 points) at the final follow-up. The preoperative median range of knee motion was 66° (range, 15° to 125°), and the median range of knee motion at the final follow-up was 97° (range, 30° to 140°).

All but 6 knees were resurfaced patella during the second or third two-stage revision TKA. No knee suffered from periprosthetic fracture. Six of the 16 knees with a third two-stage revision TKA was not able to be resurfaced due to insufficient bone stock. The remaining 10 knees had no problem related to patella.

## Discussion

The aim of this study was to determine the long-term clinical and radiographic results of the second or third two-stage revision TKA for infection using modern operative techniques and implants. The risk of failure at 15.1 years caused by recurrence of infection and mechanical reasons were approximately equal (failure rate of 5% due to mechanical future and 6% due to recurrent infection).

Patients with a previously failed two-stage revision TKA present a challenge. There is limited published data available on the second two-stage revision TKA [3, 4, 10, 16, 19–21], with average success rate reported to be 56% (range, 4–100%). Haleem *et al*. [3] reported that the survivorship free rate for implant removal for any reason was 77.3% at 10 years. Furthermore, they reported that the survivorship free rate for implant removal for reinfection was 85% at 10 years and survival free rate of revision for mechanical failure was 91% at 10 years. Backe *et al*. [10] reported no failure after a second two-stage reimplantation. Azzam *et al*. [20] reported a 78% (14 of 18 cases) success rate after a second two-stage revision TKA. Four of 18 cases failed due to recurrent infection. Furthermore, Vadiee *et al*. [21] reported an overall success rate after a second two-stage revision TKA of 74% (14 of 19 cases). Stammers *et al*. [22] suggested that following a failed two-stage revision TKA, a second two-stage revision TKA eradicated infection in 8 of 19 patients (42%). A third two-stage revision was performed in 5 of the remaining 11 patients, eradicating infection in 3, with an average follow-up of 43 months. In the current study, irrigation and open debridement with retention of the prosthesis was tried in all knees with recurrent infection after two-stage revision TKA which doomed to failure 100%. Overall success rate of salvage of the prosthesis was 78% (49 of 65 knees) after a second two-stage revision TKA. The remaining 16 knees underwent a third two-stage revision TKA and the survival rate free of implant removal was 75% (12 of 16 knees). Therefore, overall control rate of infection and survival rate free of implant removal, at a mean time of 15.1 years, in the current study was 94% (61 of 65 knees) after a second or a third two-stage revision TKA.

The most common microorganism identified in infection after a second two-stage revision TKA in this study was coagulase-negative *Staphylococcus aureus* (32%). A similar finding has been confirmed by others [21, 22]. The least favorable results in our study were observed in the patients who had methicillin-resistant *Staphylococcus aureus* and fungus infection. The difference in recurrence rate of infection between knees with methicillin-resistant *Staphylococcus aureus* and fungus and other organisms was significant (*p* = 0.01). Similar results were also observed in the aforementioned studies [9, 20, 22–25].

The strengths of this study include: (1) the relatively large number of single-surgeon patients and the long-term follow-up period; (2) the uniformity of implant designs and prosthetic fixation; and (3) the fact that this study focused on infection eradication and patient function. Our study is not without some limitations: (1) we had no comparative data on whether knee arthrodesis similarly eradicates infection or provides comparable functional results; and (2) the retrospective nature of the study may have introduced recall bias, and the review was not blinded when stratifying patient characteristics.

In conclusion, the results in the current study suggest that a second or third two-stage revision TKA is likely to result in a satisfactory outcome. In contrast, patients with methicillin-resistant *Staphylococcus aureus* and fungus infection tended to have a higher recurrence of infection after a second or third two-stage revision TKA.

## Data Availability

Data were sufficient to support the study and more studies are needed in this area for final conclusion. The data are available through PubMed.
